# Autophagy-Related Gene Signature Highlights Metabolic and Immunogenic Status of Malignant Cells in Non-Small Cell Lung Cancer Adenocarcinoma

**DOI:** 10.3390/cancers14143462

**Published:** 2022-07-16

**Authors:** Lucas Leonardi, Sophie Siberil, Marco Alifano, Isabelle Cremer, Pierre-Emmanuel Joubert

**Affiliations:** 1 Institut National de la Santé et de la Recherche Médicale (INSERM), UMRS1138, Centre de Recherche des Cordeliers, 75006 Paris, France; lucas.leonardi@sorbonne-universite.fr (L.L.); sophie.siberil@sorbonne-universite.fr (S.S.); marco.alifano@aphp.fr (M.A.); isabelle.cremer@sorbonne-universite.fr (I.C.); 2 Faculté des Sciences, Sorbonne Université, 75005 Paris, France; 3 Département de Chirurgie Thoracique, Hôpital Cochin Assistance Publique Hôpitaux de Paris, 75014 Paris, France

**Keywords:** autophagy, autophagy-gene signature, NSCLC, adenocarcinoma

## Abstract

**Simple Summary:**

The role of autophagy in lung cancers is still controversial, mainly because the visualization of autophagy levels in patients remains challenging. One interesting approach consists of studying autophagy at the transcriptomic level. In this line, many transcriptomics analyses performed on autophagy genes focused on the discovery of new biomarkers to predict the efficiency of antitumor therapies. However, the majority of these studies were based on global transcriptomic analysis of the whole tumor microenvironment, and few investigations have been performed on malignant cells themselves. The goal of this study was not to determine another new predictive signature based on autophagy-related genes. Instead, we investigated the expression of autophagy genes to understand the involvement of this process in lung cancer homeostasis. Specifically, we discovered a new autophagy signature that correlates with the metabolic and immunogenic status of malignant cells, supporting the relationship between autophagy and tumor growth in lung cancer patients.

**Abstract:**

Autophagy is a self-degradative mechanism involved in many biological processes, including cell death, survival, proliferation or migration. In tumors, autophagy plays an important role in tumorigenesis as well as cancer progression and resistance to therapies. Usually, a high level of autophagy in malignant cells has been associated with tumor progression and poor prognostic for patients. However, the investigation of autophagy levels in patients remains difficult, especially because quantification of autophagy proteins is challenging in the tumor microenvironment. In this study, we analyzed the expression of autophagy genes in non-small cell lung (NSCLC) cancer patients using public datasets and revealed an autophagy gene signature for proliferative and immune-checkpoint-expressed malignant cells in lung adenocarcinoma (LUAD). Analysis of autophagy-related gene expression profiles in tumor and adjacent tissues revealed differential signatures, namely signature A (23 genes) and signature B (12 genes). Signature B correlated with a bad prognosis and poor overall and disease-specific survival. Univariate and multivariate analyses revealed that this signature was an independent factor for prognosis. Moreover, patients with high expression of signature B exhibited more genes related to proliferation and fewer genes related to immune cells or immune response. The analysis of datasets from sorted fresh tumor cells or single cells revealed that signature B is predominantly represented in malignant cells, with poor expression in pan-immune population or in fibroblast or endothelial cells. Interestingly, autophagy was increased in malignant cells exhibiting high levels of signature B, which correlated with an elevated expression of genes involved in cell proliferation and immune checkpoint signaling. Taken together, our analysis reveals a novel autophagy-based signature to define the metabolic and immunogenic status of malignant cells in LUAD.

## 1. Introduction

Lung tumors are among the most common in the world and are one of the leading causes of cancer deaths worldwide [1]. Among them, NSCLC accounts for 85% of all lung tumor cases and it is mainly composed of lung squamous cell carcinoma (LUSC) and adenocarcinoma (LUAD) [1]. Although the available therapeutic arsenal, including surgical procedures, chemotherapy, targeted molecules and immunotherapy, have allowed undeniable progress in lung cancer treatment, the 5-year survival rate of NSCLC patients remains unsatisfactory. This relative inefficiency is mainly due to the lack of information about the tumor microenvironment during treatment decision. For this reason, many investigations have focused on the discovery of a new prognostic assessment method to help individualized treatment of NSLCL patients [2].

Autophagy is a cellular process associated with the prevalence and progression of lung cancer [3,4]. Autophagy is a conserved catabolism pathway that plays a key role in the maintenance of cellular homeostasis. It is a multistep mechanism, consisting of the formation of the phagophore that elongates and engulfs targeted proteins or organelles in a double-membrane vesicle called the autophagosome, and finally fuses with late endosomes and/or lysosomes [5]. This process is orchestrated by a large variety of proteins, including the autophagic proteins (Atg), organized in complexes. Autophagy induction is modulated by two protein complexes, the ULK1/2 (unc51-like autophagy activating kinase) and the Beclin-1/PI3KC3 (class III phosphatidylinositol 3-kinase) complexes. Once activated, these complexes recruit other proteins involved in the elongation and formation of autophagosomes, including the two conjugated systems Atg12-Atg5-Atg16L and LC3. After completion, the mature autophagosome fuses with lysosomes to form autolysosomes, wherein the sequestered materials and organelles are degraded by lysosomal enzymes [6]. Then, the degradation products are recycled for cell synthesis biological processes. Autophagy is one of the most important survival mechanisms under stress conditions and is involved in cellular homeostasis and proliferation [5]. Several studies have demonstrated links between autophagy and carcinogenesis, highlighting a dual role for autophagy in cancer. Depending on the tumor model and/or tumor state, autophagy may have pro- or anti-tumor effects. In the initial stage of cancer, autophagy protects normal cells from tumorigenesis by preventing DNA damages and mutations [3]. In established solid tumors, autophagy has been shown to favor tumor development by enhancing tumor growth, cell survival, resistance to platinum-based chemotherapy and metastasis formation [7]. Autophagy may also interfere with immunotherapy, since some studies showed a link between autophagy and immune checkpoint activity and/or expression, including CTLA-4, IDO and PD1/PD-L1 [8,9]. Autophagy also has a critical function in tumor immune cells and tumor immune response, promoting the immunogenic cell death of tumor cells and favoring immune cell activation and proliferation [10]. Meanwhile, autophagy in cancer-associated fibroblasts (CAFs) promotes tumorigenesis by providing nutrients to the cancerous cells and by favoring epithelial to mesenchymal transition, angiogenesis and stemness [11].

Many transcriptomic analyses performed on autophagy genes have focused on the discovery of new biomarkers to predict the efficiency of anti-tumor therapies and to guide individualized treatment in NSCLC patients [12,13,14,15,16]. However, the majority of these studies are based on global transcriptomic analysis of the whole tumor microenvironment, and few investigations have been carried out on malignant cells themselves. Regarding the global effect of autophagy on cells infiltrating the tumor microenvironment, it is important to determine a signature to identify the functional status of each cell type. In this study, we explore the relationship between 232 autophagy-related genes and biological pathways related to tumor progression in multiple LUAD datasets. Comparing tumors with adjacent tissue, we identified two signatures composed of twenty-three (signature A) and twelve (signature B) genes, and these signatures were correlated with survival, tumor metabolic status and immunology factors in LUAD patients. RNA sequence profiling of flow-sorted malignant cells, endothelial cells, immune cells and fibroblasts from freshly resected primary human NSCLC reveals that signature B was mainly expressed by malignant cells. The predominant expression of signature B in malignant cells was validated in the single cell sequencing data analysis. Deeper investigations supported the correlation between autophagy with tumor cell proliferation and immune checkpoint expression in malignant cells, highlighting the impact of autophagy in tumor cell progression and its potential role in immunotherapy. Therefore, our study provides a new autophagy-related signature that predicts the biological status of malignant cells in LUAD patients.

## 2. Materials and Methods

### 2.1. Dataset Source, Pre-Processing and Workflow

**Figure 1:**
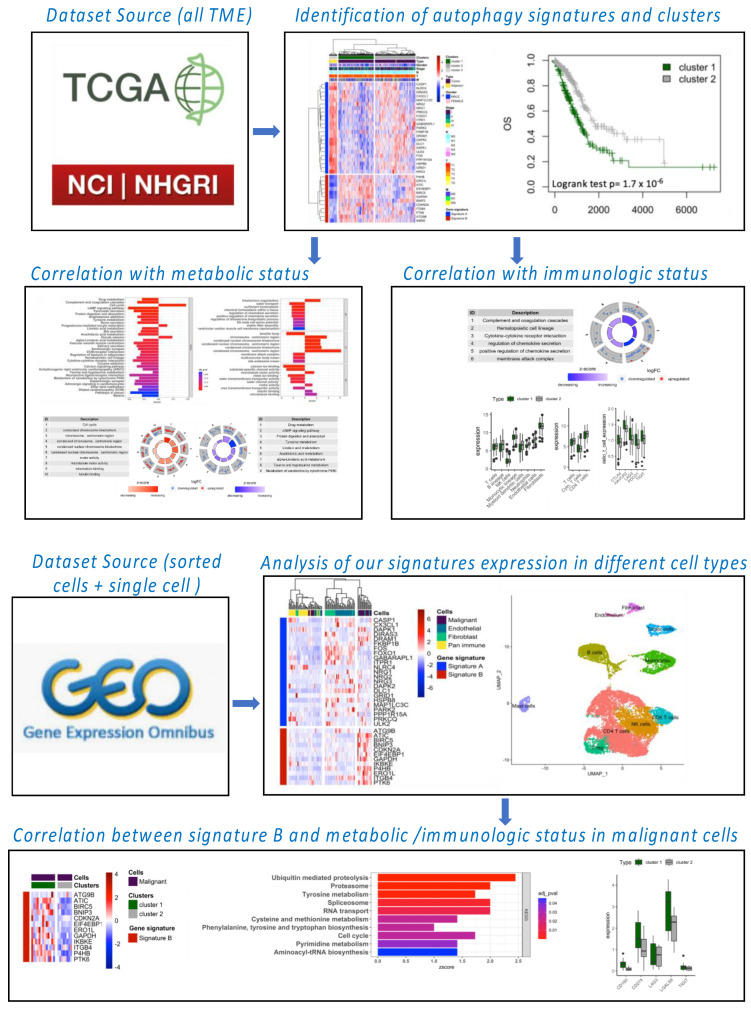
Workflow of bioinformatics analysis. Datasets were obtained from TCGA and GSO databases. Differential expression analysis was performed for 232 autophagy genes (human autophagy database) between tumor and adjacent tissue, and clusters of patients were examined in terms of survival and other clinical features. Correlations between autophagy clustering and metabolism or immunologic pathways were studied. The autophagy signature was studied in flow-sorted malignant, endothelial, immune and fibroblast cells from freshly resected primary human NSCLC and in single-cell sequencing data, showing a predominant expression of signature B in the malignant cell population. The correlation between autophagy clustering and metabolic or immunological status was confirmed for malignant cells.

The workflow of our bioinformatic analysis is summarized in Figure 1. LUNG and LUAD gene expression datasets and associated clinical information were obtained from The Cancer Genome Atlas (TCGA) and Gene-Expression Omnibus (GEO) databases. The download gene expression profiles from TCGA met the following conditions: (1) the primary site was “bronchus and lung”; (2) the program was “TCGA”; (3) the disease type was “adenomas and adenocarcinomas” and/or “squamous cell neoplasms”; (4) the data category was “transcriptome profiling”; (5) the data type was “Gene Expression Quantification”; and (6) the workflow type was “HTSeq-FPKM”. TCGA-LUNG samples used for this analysis included 110 normal samples and 1019 tumor samples and TCGA-LUAD included 59 normal samples and 517 tumor samples. In addition, we downloaded series matrix files and platform files of four datasets, including one for global TME (GSE31210), one for RNA sequence profiling of flow-sorted malignant cells, endothelial cells, immune cells and fibroblasts from resected primary human NSCLC (GSE111907), one for single-cell analysis (GSE123904) and one for A549 cells invalidated by siRNA for atg5 and ulk1 genes involved in the autophagy process (GSE73158). The basic information regarding all databases is provided in Table 1.

The fragments per kilobase million (FPKM) values were converted into the transcripts per million (TPM) data using the R package “limma”. R (version 4.0.3, R core team, https://www.r-project.org, accessed on 5 June 2022) was used to process data. Processing for GSE datasets will be explained in the appropriate section.

### 2.2. Autophagy Signature and Clustering Analysis

We used the human autophagy database (HADd) to analyze the differential expression of autophagy-related genes (*n* = 232) between normal and tumor tissue. Differentially expressed genes were based on logFC > 1 or <−1 and adjusted *p*-value < 0.05 using “limma” package in R. LUNG or LUAD samples were grouped into clusters according to their expression of autophagy signature genes (genes differentially expressed between adjacent and tumor samples). Kaplan–Meier survival curves were applied to each cluster and log-rank tests were performed to compare the overall survival (OS) and disease-specific survival (DSS) between clusters. Univariate and multivariate Cox analysis was performed to analyze the hazard ratio of clusters. Similar analysis was performed for individual genes of autophagy signature.

### 2.3. Functional Annotation Enrichment

To determine the variation of biological pathways between clusters, the differential expression analysis of whole genome between two clusters was performed. Differentially expressed genes were based on logFC > 1 or <−1 and adjusted *p*-value < 0.05 using “limma” package in R. Based on DEG analyses, gene set enrichment analysis (GSEA) and Gene Ontology (GO) enrichment analysis were performed using the “Enrichr” website (https://maayanlab.cloud/Enrichr/, accessed on 5 June 2022) and the results were plotted using the “GOplot” R package. We selected the function and pathways with a strict *p*-value < 0.05. For “circle plot”, we selected important pathways or functions involved in anabolism or catabolism.

### 2.4. Immune Cell Infiltration, Stromal Cell Population and Exhaustion Marker Expression Analysis

For immune cell infiltration and the stromal cell population, we applied the microenvironment cell population-counter (MCP-count) method [17]. A total of 10 cell signatures were calculated to determine T cells, cytotoxic T cells, CD4+ T cells, B cell lineage, NK cells, monocyte lineage, myeloid dendritic cells, neutrophils, endothelial cells and fibroblasts. To determine the expression of exhaustion markers in T cells, we calculated the expression of *CTLA-4*, *HAVCR2*, *LAG3*, *PDCD1* and *TIGIT* genes according to the median of T-cell expression in TCGA.

### 2.5. Autophagy Signature Expression in Sorted-Cell Fresh Tumor Samples Datasets and Single Cell Analysis

RNA sequence profiling of flow-sorted malignant cells (EPCAM^+^ CD45^−^ CD31^−^), endothelial cells (CD31^+^ CD45^−^ EPCAM^−^), immune cells (CD45^+^ EPCAM^−^) and fibroblasts (CD10^+^ CD45^−^ EPCAM^−^ CD31^−^ CD10^+^) from freshly resected primary human NSCLC (GSE111907 dataset) were used to calculated expression levels of autophagy signature A and B on different cell populations. For these analyses, we only selected adenocarcinoma subtypes. We analyzed 21 malignant samples, 22 pan-immune samples, 23 endothelial samples and 22 fibroblasts samples. Samples were clustered according to their expression of autophagy signature gene and data were visualized by “pheatmap” R package. For single-cell analysis, we utilized GSE123904 datasets. We used only primary tumor samples for this analysis (LX653, LX661, LX675, LX676, LX678, LX680, LX682 and LX684). A total of 18,124 cells were analyzed. Single cell clustering and dimension reduction were performed by R package “Seurat”. The principal component analysis (PCA), “FindNeighbors” and “FindClusters” packages were employed to construct the cell culturing. The “UMAP” package was used to visualize data, and we utilized the “FeaturePlot” function form “Seurat” to visualize the expression of the autophagy signatures. Cell clusters were annotated according to the gene expression in each cluster revealed by the “FindAllMarkers” function of “Seurat” package.

### 2.6. Autophagy Clustering Analysis in Malignant Tumor Cells

We selected 22 malignant samples (adenocarcinoma) from the GSE111907 dataset and samples were clustered according to their expression of the autophagy signature B. Gene Set Enrichment Analysis (GSEA) was performed as previously described.

### 2.7. Gene Set Enrichment Analysis in A549 Deficient for Autophagy

To examine the impact of autophagy genes in global biological processes, we used GSE73158 datasets. We selected three lung adenocarcinoma cell lines, A549 treated with siRNA against *ATG5*, A549 treated with siRNA against *ULK1*, and their respective siRNA control. We performed differential expression analysis for siRNA-treated cells according to their respective control and selected upregulated (logFC > 1) or down-regulated (logFC < −1) genes. To perform functional annotation enrichment analyses, we chose genes which were significantly modified in both si*ATG5*- and si*ULK1*-treated cells.

### 2.8. Cell Culture, Proliferation Analysis and Confocal Microscopy

The human lung adenocarcinoma A549 cell line and the murine adenocarcinoma LLC and carcinoma KP cell lines were cultured in DMEM F-12 medium (Gibco, Waltham, MA, USA) supplemented with 10% FBS (Eurobio Scientific, Ulis, France), 1% non-essential amino acid (Gibco), 1% herpes (Gibco), 1% glutamate (Gibco) and 1% Na^+^/pyruvate (Gibco) in a standard 5% CO_2_ incubation atmosphere at 37 °C. The human lung squamous cell carcinoma SK-MES cell line was cultured in EMEM F-12 medium (Gibco) supplemented with 10% FBS (Eurobio Scientific), 1% non-essential amino acid (Gibco), 1% 1% herpes (Gibco), 1% glutamate (Gibco) and 1% Na^+^/pyruvate (Gibco) in a standard 5% CO_2_ incubation atmosphere at 37 °C.

For in vitro proliferation assays, 150,000 cells were stained with CFSE (1/500, ThermoFischer, Waltham, MA, USA) for 30 min at 37 °C in PBS and plated in the 6-well plate for 24 h. Cells were cultured for 24, 48, 72 and 96 h in the presence or not of 10 mM of 3-methyladenin (Sigma, Saint-Louis, MO, USA), 100 nM of wortmannin (Sigma), 10 µM of SAR405 (MedChemExpress, Monmouth Junction, NJ, USA), or 100 nM of bafilomycin (Sigma), and stained with live/dead kit (1/100, near-IR, ThermoFisher). Analysis of CSFE staining was performed using the BD LSR Fortessa Cell analyzer. Flow cytometry data were analyzed by FlowJo software.

For in vitro analysis of the autophagy level, 35,000 A549 cells expressing the GFP-LC3 protein were plated in a 24-well plate containing coverslips for 24 h. Cells were then cultured for 24 h in the presence or not of 10 mM of 3-methyladenin (Sigma), 100 nM of wortmannin (Sigma), 10 µM of SAR405 (MedChemExpress), or 100 nM of bafilomycin (Sigma), and were mounted on the slides using glycergel (Dako, Santa Clara, CA, USA). The autophagosomes were observed by confocal microscopy (LSM 710) and enumerated by a personal R script.

### 2.9. Statistical Analysis

R software (v4.0.3) was used for all bioinformatic statistical analyses, and PRISM software was employed for in vitro experiments. The Wilcoxon test was used to compare the differences between the two groups. The Kruskal–Wallis test was utilized to compare the differences between three groups and above. The survival time of the patient was evaluated by Kaplan–Meier survival analysis, and the different groups were compared by utilizing a log-rank test. Univariate and multivariate Cox regression analysis was used to investigate the independent prognostic factor, employing the “survival” R package. The Benjamin–Hochberg method was used to calculate *p*_value for FRDs conversation and DEG analyses. Single-cell analysis was performed using R package “Seurat”. Survival curves were performed utilizing R package “survminer”. All heatmaps were generated by R package “pheatmap”. We employed “GOplot” R package to visualize the functional annotation enrichment analyses. Data visualization was performed using R package “ggplot2”. The R packages utilized in this study could be obtained from “bioconduction”.

## 3. Results

### 3.1. Autophagy Gene Expression Was Distinct, According to the Subtype of Lung Tumors

To investigate the impact of autophagy genes in lung tumors, we analyzed the differential expression of autophagy genes (from the human autophagy database) in tumors (*n* = 1019) versus adjacent (*n* = 110) tissues using TCGA-LUNG public cohorts. Among the autophagy-related genes tested (*n* = 232), 23 genes were down-regulated and 16 were upregulated in tumors as compared to the adjacent tissue (Figure S1A). Further analysis revealed a clear separation of patients into two groups, with 86% of adenocarcinoma in one group and 96% of squamous-cell carcinoma in the other group, suggesting that patients with different histology subtypes expressed very distinct autophagy genes (Figure S1B,C). No differences were observed according to gender, stage of cancer or TNM classification in the two groups of patients (Figure S1B). Given that the autophagy signature is very different according to the tumor subtype, it is important to separately analyze adenocarcinoma and squamous cell carcinoma in this context. In this study, we focused our research on adenocarcinoma subtypes.

### 3.2. Autophagy-Related Gene Signature in Lung Adenocarcinoma Correlates with an Increase in Anabolic and a Decrease in Catabolic Pathways

**Figure 2:**
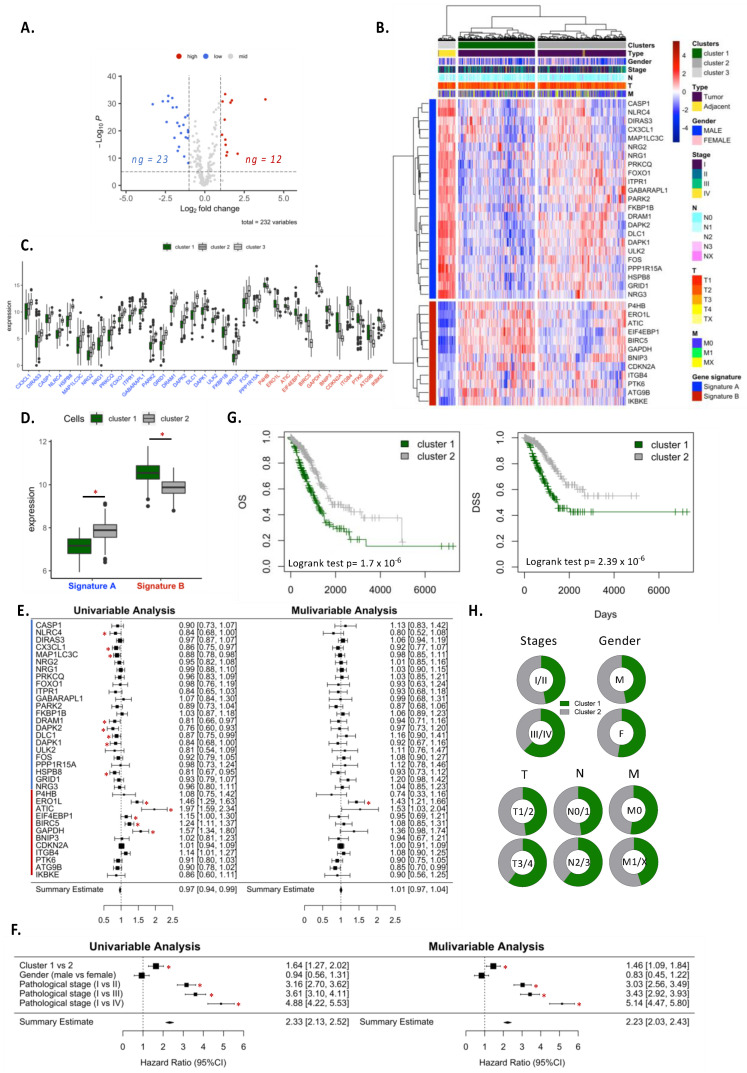
Autophagy genes screening and cluster analysis in lung adenocarcinoma. (**A**) Differential expression analysis for the human autophagy database (*n* = 232 genes) of TCGA-LUAD patients between tumor (*n* = 517) and adjacent tissue (*n* = 59). (**B**,**C**) Thirty-five differentially expressed genes of autophagy in TCGA-LUAD datasets. (**D**) Expression of signature A and B in clusters 1 and 2 in TCGA-LUAD datasets. (**E**,**F**) Autophagy signature gene expression, clustering of patients, clinicopathological and OS of univariate and multivariate Cox regression analysis in the TCGA-LUAD datasets. (**G**) Kaplan–Meier OS and DSS curve in the TCGA-LUAD dataset. (**H**) Distribution analysis of clusters according to stages, gender and TNM classification in the TCGA-LUAD datasets. *, *p*-value < 0.05.

Using the TCGA-LUAD cohort, we performed a similar experiment to that previously described by comparing the autophagy gene expressions between tumor (*n* = 517) and adjacent tissue (*n* = 59). Twenty-three genes were down-regulated and 12 were up-regulated in tumor samples as compared with the adjacent tissues (Figure 2A). Two clusters of patients differentially expressed autophagy genes (Figure 2B). Patients in cluster 1 comprised the most distinct cluster for autophagy gene expression as compared with the adjacent tissue (Figure 2C). Moreover, cluster 1 expressed significantly more up-regulated genes (called signature B) and fewer down-regulated genes (called signature A) as compared to cluster 2 (Figure 2D). Univariable analysis for the hazard ratio revealed that some genes involved in signature A (*NLRC4*, *CX3CL1*, *MAP1LC3C*, *DRAM1*, *DAPK2*, *DLC1*, *DAPK1* and *HSPB8*) were associated with a good prognosis (Figure 2E). In contrast, some genes in signature B (*ERO1L*, *ATIC*, *EIF4EBP1*, *BIRC5* and *GAPDH*) were associated with a bad prognosis. Interestingly, cluster-related autophagy genes were an independent factor significantly associated with a bad prognosis (Figure 2G). These observations were supported by the analysis of the overall survival (OS) and disease-specific-survival (DSS), showing that patients in cluster 1 have a lower survival rate as compared to those in cluster 2 (Figure 2G). Of note, while no distinction has been found according to gender, stage or TNM classification, patients in advanced stages (stage III/IV or T3/4 or N2/3) were more represented in cluster 1 than cluster 2 (Figure 2H).

**Figure 3:**
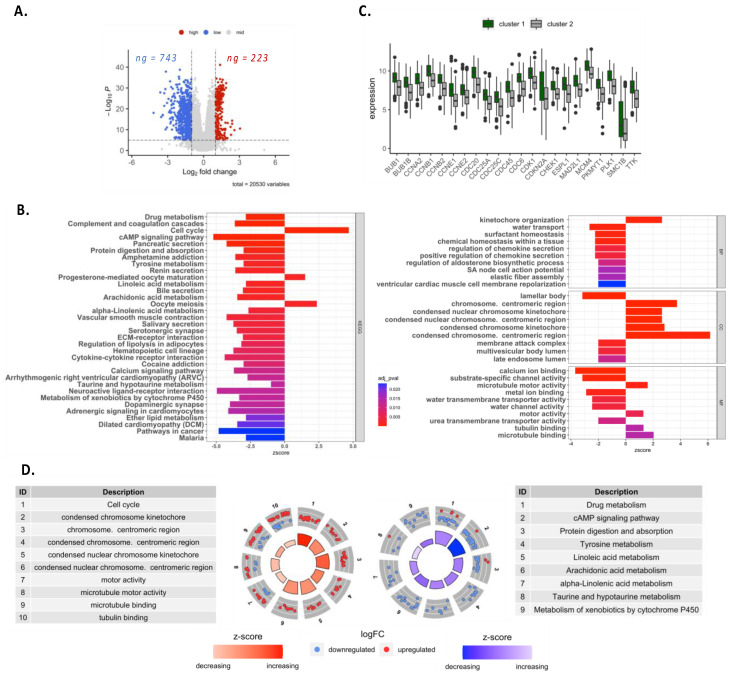
Differential gene expression and enrichment analysis for clusters. (**A**) Volcano plot for differential expression analysis in all genomes (cluster 1 versus cluster 2) in the TCGA-LUAD datasets. (**B**) Differential gene GO and KEGG enrichment analysis, BP stands for biological processes, CC stands for cellular components, and MF stands for molecular function. (**C**) Significant expression of genes involved in cell cycle pathways. (**D**) Representation for gene expression in GO and KEGG enrichment analysis for anabolism (left) and catabolism (right) pathways.

To further investigate the implication of autophagy gene signatures in lung adenocarcinoma, we performed an analysis of the differential expression of all the genes between cluster 1 and cluster 2 and observed that 743 genes were down-regulated and 223 were up-regulated in cluster 1 as compared to cluster 2 (Figure 3A). KEGG and GO analyses showed that many biological pathways were significantly impacted by the differential gene expression profiles between the two clusters (Figure 3B). Among these pathways, several genes implicated in anabolism pathways were increased in cluster 1 as compared to cluster 2, including genes involved in cell proliferation (e.g., “Cell cycle” or pathways related to chromosome or microtubule activity) (Figure 3B–D). On the contrary, genes implicated in catabolic processes were globally decreased in cluster 1, including genes involved in “drug metabolism”, “cAMP signaling pathways” or “Protein digestion and absorption” (Figure 3D). These results suggest that in cluster 1, cellular metabolic processes are more activated than in cluster 2, with potentially a higher rate of cellular proliferation.

### 3.3. Autophagy-Related Gene Signature Highlighted a Decrease in Immunity-Related Pathways and an Increase in Exhaustion Genes

**Figure 4:**
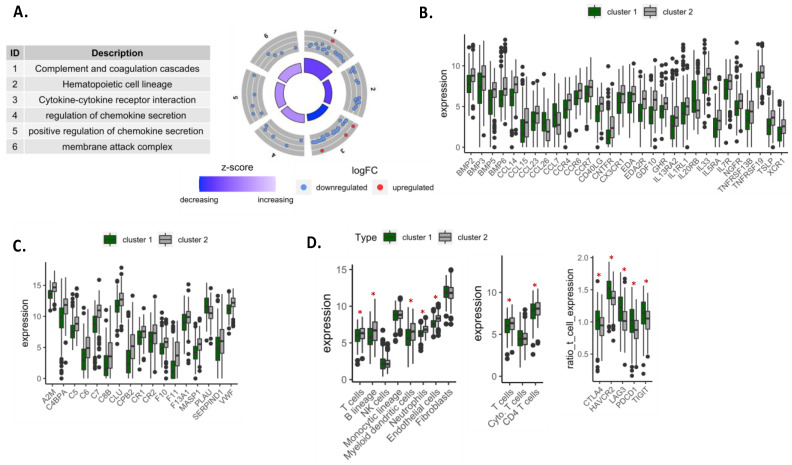
Enrichment analysis related to immune pathways. (**A**) Representation for gene expression in GO and KEGG enrichment analysis related to immunity. (**B**,**C**) Significant expression of genes involved in cytokine-cytokine receptor interaction and complement cascade, respectively. (**D**) The expression of genes related to immune cells according to MCP counter database in the TCGA-LUAD datasets. *, *p*-value < 0.05.

Besides metabolic pathways, some genes involved in immunity were also differentially expressed between cluster 1 and cluster 2 (Figure 3B). Indeed, a decreased expression of genes involved in the complement cascade, hematopoietic cell lineage and cytokine/chemokine pathways was observed in cluster 1 as compared to cluster 2 (Figure 4A–C), suggesting that patients in cluster 1 exhibited a poorly infiltrated tumor microenvironment. To support this result, we evaluated immune cell infiltration in each cluster using the MCP counter method [17]. We observed a significant decrease in genes related to T and B lymphocytes and myeloid dendritic cells, neutrophils and endothelial cells in cluster 1 as compared to cluster 2 (Figure 4D). Further analysis revealed that patients in cluster 1 expressed more genes involved in T-cell exhaustion (*CTLA-4*, *HAVCR2*, *LAG3*, *PDCD1* and *TIGIT*) (Figure 4D. Taken together, our results show that patients with high signature B and low signature A expression (cluster 1) exhibited active cell proliferation but a reduction in immune response, which is consistent with the poor survival rate observed for patients in clusters 1.

To investigate whether the correlation between autophagy signature and metabolic status or immune cell infiltration could be influenced by the stage of the cancer, we performed similar analyses in LUAD-TCGA datasets stratified by tumor stages (stages I/II or stages III/IV). We observed a very similar clustering for patients in both early and advanced stages (with around 90% of similarity for differential expressed genes in the early stages and 85% in the advanced stages) (Figures S2A and S3A). Patients in cluster 1 comprised the most distinct cluster for autophagy gene expression as compared to adjacent tissue. The overall survival (OS) and disease-specific survival (DSS) analyses revealed that patients in cluster 1 have a lower survival rate as compared to those in cluster 2 in the early stages of the tumor (Figure S2B). In more advanced cancers, patients in cluster 1 have a lower DSS, but no significant difference was observed for OS (Figure S3B). For both groups of tumor stages, the KEGG and GO analyses showed that patients in cluster 1 expressed more genes involved in cell proliferation and fewer genes implicated in catabolic processes (Figures S2C and S3C). Patients in cluster 1 were also less infiltrated by immune cells and expressed more genes involved in T-cell exhaustion as compared to patients in cluster 2 (Figures S2D and S3D). These data showed that our autophagy gene signatures correlate with metabolic status and immune infiltration independently of the tumor stages.

Interestingly, we confirmed a correlation between autophagy-related gene signatures and metabolic and immunologic status using another cohort of adenocarcinoma. Applying autophagy signatures in 226 LUAD patients (GSE31210 datasets), we defined two clusters of patients. According to our previous observation, the cluster with a low expression of signature A and high expression of signature B exhibited the worst prognostic value, the most active metabolic status and lowest immune cell infiltration (Figure S4A–D).

### 3.4. The Autophagy Signature B Was Enriched in Malignant Cells and Revealed Metabolic and Immunogenic Status of Tumor Cells

**Figure 5:**
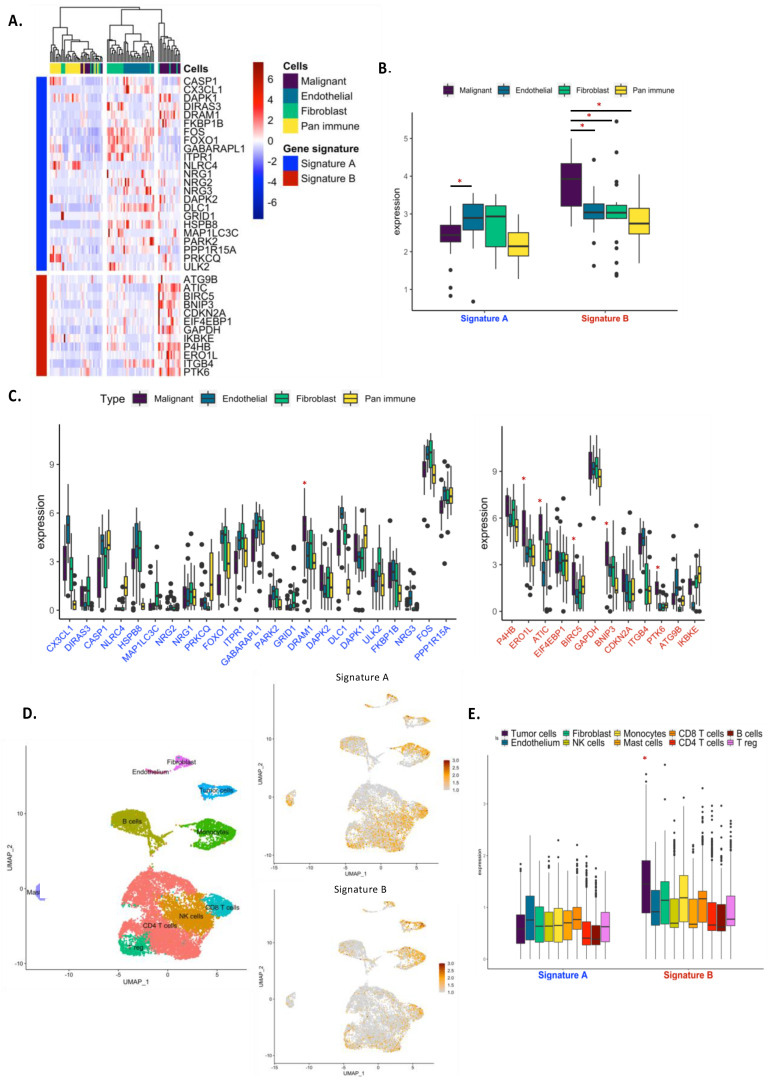
Expression of autophagy-related signatures in different subtypes of TME. (**A**) Unsupervised clustering of cell types using autophagy gene signatures in the GSE111907 datasets. (**B**,**C**) Expression of autophagy gene signatures according to cell types in the GSE111907 datasets. (**D**,**E**) Single-cell analysis of autophagy gene signature expression in the GSE123904 datasets. *, *p*-value < 0.05.

Using a cohort of adenocarcinoma from GSE (GSE111907), we compared the expression of autophagy gene signatures between tumor-infiltrating cell subsets. In this cohort, RNA-seq profiling of flow-sorted malignant cells, endothelial cells, immune cells and fibroblasts from resected primary human NSCLC was performed. While the global autophagy gene expression was not different between cell subtypes (Figure S5A, Table S1), analyses of our autophagy-related signature revealed that signature B was significantly more expressed in malignant cells as compared to immune, fibroblast or endothelial cells (Figure 5A,B). Signature A was preferentially expressed in fibroblasts and endothelial cells, with a low expression in malignant cells (Figure 5B and Table S2). One gene in signature A (*DRAM1*) and five genes in signature B (*ERO1L*, *ATIC*, *BIRC5*, *BNIP3* and *PTK6*) were significantly more expressed in malignant cells as compared to other cell subtypes (Figure 5C and Table S3). These results were supported using the single-cell transcriptional landscape of primary lung adenocarcinoma (GSE123904) (Figure 5D and Figure S5B and Table S4). Cells were clustered using weighted nearest neighbor analysis (Seurat) and clusters were annotated according to gene expression (as explained in the Materials and Methods). We then analyzed the expression of autophagy signatures in each cell and observed that tumor cells poorly express genes in signature A and highly express genes in signature B as compared to other cell types (Figure 5E and Table S5).

**Figure 6:**
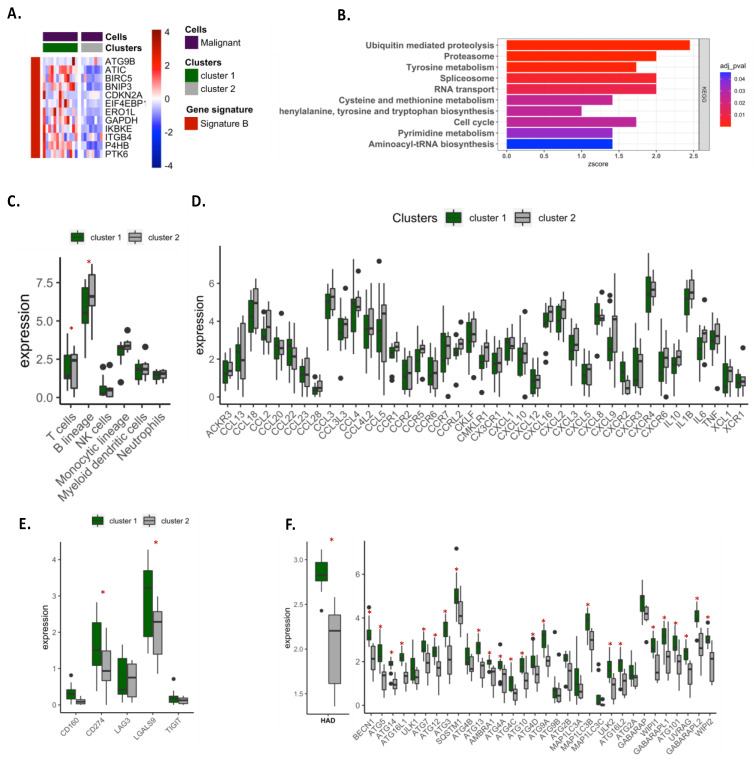
Metabolic and immunologic status of malignant cells according to signature B. (**A**) Unsupervised clustering of patients using autophagy signature B in malignant cells samples using the GSE111907 datasets. (**B**) KEGG enrichment analysis between cluster 1 and cluster 2. (**C**–**E**) Analyses of immune cell infiltration (**C**), expression of chemokine-related genes (**D**) and expression of genes involved in immune checkpoint (**E**) in clusters. (**F**) Expression of global (human autophagy database) and autophagy genes in the GSE111907 datasets. *, *p*-value < 0.05.

Clustering patients (from GSE111907 dataset) for the expression of autophagy signature B in malignant cells also revealed two clusters, with patients in cluster 1 exhibiting more genes in signature B than patients in cluster 2 (Figure 6A). As previously observed for the analysis of the global tumor microenvironment (TCGA dataset), differential gene expression analysis focusing on malignant cells revealed that patients in cluster 1 exhibited more genes involved in cell proliferation and/or anabolism as compared to patients in cluster 2 (Figure 6B). Comparing immune cell infiltration between clusters revealed that patients in cluster 1 were less infiltrated by T and B cells and expressed fewer chemokine-related genes in malignant cells as compared to cluster 2 (Figure 6C,D). Moreover, malignant cells of cluster 1 expressed more *CD274* (PD-L1) and *LGALS9* (galectin 9) genes (Figure 6E). Together, these data suggest that malignant cells expressing high levels of signature B were more prone to proliferating and inducing immune suppression. Interestingly, expression analysis of autophagy genes (from the human autophagy database) showed that cluster 1 exhibited a higher autophagy level as compared to cluster 2 (Figure 6F), underlining a close relationship between the metabolic and immunogenic status of malignant cells and autophagy.

### 3.5. Autophagy Is Required for the Proliferation of Tumor Cells

**Figure 7:**
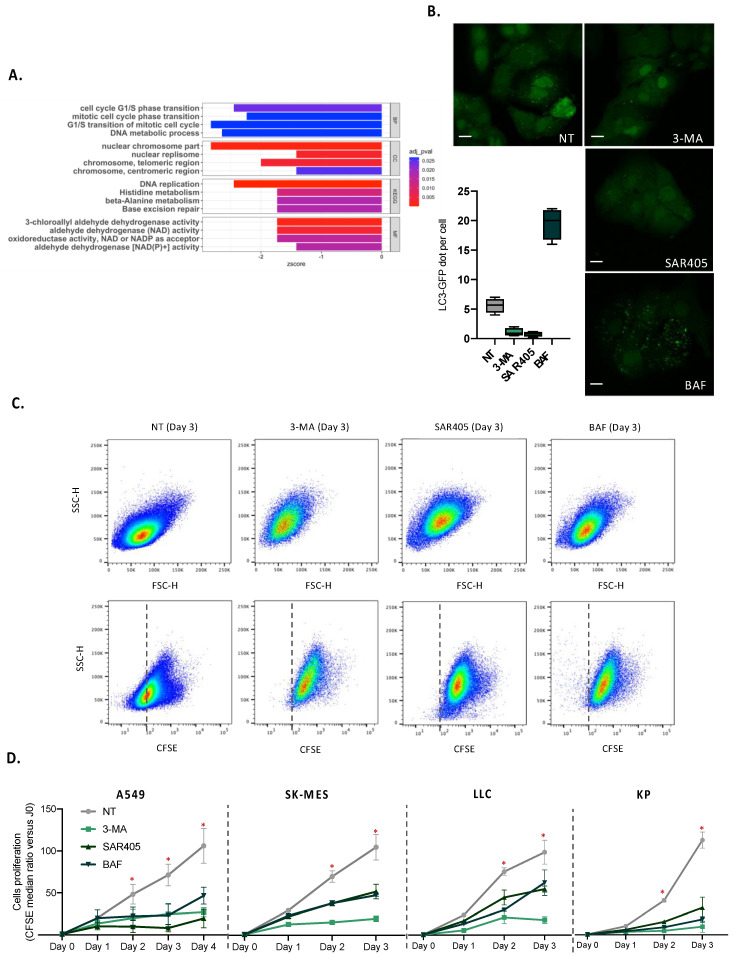
Impact of autophagy in tumor cell proliferation. (**A**) Differential gene GO and KEGG enrichment analysis in GSE73158 datasets (A549 control versus autophagy deficient), BP stands for biological processes, CC stands for cellular components, and MF stands for molecular functions. (**B**) A549-GFP-LC3 cell lines were treated or not with 3-methyladenine (3-MA), SAR405 or bafilomycin (BAF) and the number of autophagosome was evaluated by confocal microscopy. Scale bars represent 10 µm. (**C**) A549 cell lines were cultured in media containing CFSE and were treated or not with 3-methyladenine (3-MA), SAR405 or bafilomycin (BAF) for 3 days. Morphological modification and CFSE staining were analyzed by flow cytometry. (**D**) A549, SK-MES, LLC and KP cell lines were treated as previously described and the proliferation of cells was studied following the CFSE staining. *, *p*-value < 0.05.

To demonstrate the impact of autophagy in lung tumor cell expansion, we first compared gene expression in A549 adenocarcinoma cell lines in which autophagy genes (*ATG5* or *ULK1*) had been deleted or not (GSE73158 dataset) (Figure 7A). The deletion of *ATG5* or *ULK1* induced a decreased expression of genes involved in the cell cycle and/or replication, supporting the suggestion that autophagy is required for tumor cell expansion. To confirm this result, we performed in vitro analysis assay for tumor cells proliferation. The inhibition of the autophagy machinery significantly impaired the autophagy level as expected, decreasing the autophagosome number when the initiation of autophagy was inhibited (cells treated with 3-Methyladenin or SAR405) and increasing the accumulation of the autophagy vacuoles when the maturation step was blocked (cells treated with bafilomycin) (Figure 7B). The cell proliferation of several lung tumor cell lines was drastically decreased when autophagy was inhibited for both the initiation and maturation steps (Figure 7C,D). Taken together, these results demonstrate a strong involvement of autophagy in malignant cell proliferation and metabolism, and support the relevance of using transcriptomic analysis for autophagy genes to analyze the metabolic status of malignant cells.

## 4. Discussion

As a central process of self-digestion and stress adaptation, autophagy has a remarkable impact on tumor development [4,7,18]. It can provide nutrients for cancer cell survival, proliferation and migration, promotes drug resistance and helps tumor cells to evade immune surveillance [4]. In lung cancers, several studies showed that autophagy promotes tumor cell growth and resistance to radiation or chemotherapy [19,20]. However, due to the difficulty of visualizing and quantifying autophagy in tumor patients, the role of autophagy in NSCLC patients is still unclear [20,21].

Gene expression analysis appears to be a relevant approach to analyze autophagy in NSCLC patients. We first conducted our analysis in LUAD and LUSC, which accounted for the majority of NSCLC. Based on the TCGA database, our preliminary exploration revealed that modification for the expression of autophagy genes can be observed in tumor samples, demonstrating that autophagy is particularly active in cancers. Autophagy expression was very dependent on cancer subtypes, and clear clustering of patients has been found between LUAD and LUSC samples. To further investigate autophagy in lung cancers, we focused our analysis on LUAD, which is holding the predominant position among all the pathological types of lung cancer. Performing differential expression analysis of 232 autophagy-related genes between tumor and adjacent tissue samples, we observed two clusters of patients according to the expression of autophagy signature A (23 genes) and signature B (12 genes). Patients in cluster 1, characterized by lower expression of signature A and higher expression of signature B than cluster 2, had the worst overall survival (OS) and disease-specific survival (DSS). Univariate and multivariate COX analyses suggested that autophagy signatures could be an independent feature associated with bad prognosis in patients. We showed that cluster 1 was more metabolically active, expressing anabolism-related genes involved in cell proliferation and migration. On the contrary, cluster 2 exhibited an antiproliferative phenotype, with active catabolism pathways. In addition, cluster 1 samples were less infiltrated by immune cells than cluster 2 and exhibited decreased immune response features. Analyses of autophagy signatures expression in single cells or sorted-cell datasets revealed that signature B was largely expressed by malignant cells, while signature A was preferentially expressed in endothelial and in less extend in fibroblast cells. Signature B expression in malignant cells correlated with an active metabolic feature, a decrease in immune cell infiltration and an increase in the immune checkpoint expression on tumor cells (e.g., *PD-L1* and *Galectin-9*).

Interestingly, signature B correlated with an active autophagy process, supporting the central role for autophagy in tumor proliferation and migration and suggesting an important impact of this process on immune escape.

Previous studies analyzed the expression of autophagy genes in lung tumors and constructed the autophagy-related signature to anticipate the prognosis of LUAD or LUSC patients using the TCGA datasets [15,16,22]. Two studies also determined predictive signatures based on autophagy-associated long non-coding RNAs [14,23]. The goal of this study was not to determine a new predictive signature based on autophagy-related genes. Instead, we investigated the expression of autophagy genes to understand the involvement of this process in lung tumor homeostasis. While previous studies established autophagy signatures in the global TME and correlated the expression of autophagy genes with the survival probability, we focused our analysis on malignant cells, highlighting a new autophagy signature that could help to understand the metabolic and immunologic status of these cells.

We take advantage of sorted cells and single cell datasets to analyze the expression of our signature and showed that signature B correlated with active metabolic status of tumor cells. Interestingly, the patients with high expression of signature B expressed much higher expression of autophagy genes involved in core machinery, including genes involved in the initiation complex (e.g., *BECN1*, *ULK1*, *AMBA1*) or elongation system (e.g., the majority of ATG genes, *MAP1LC3A*, *MAP1LC3B*, *MAP1LC3C*). These data supported previous studies that described a pro-tumor function for autophagy in lung cancers, favoring the proliferation and migration of tumor cells [3,20,23,24].

Our data also revealed an important impact of autophagy on immune escape, describing that autophagy gene expression can reflect the immune cell infiltration and/or the immunogenic status of malignant cells. Interestingly, the correlation between autophagy genes expression and immune infiltration has also been described in other types of tumors [23,25]. While some studies observed a similar correlation between autophagy and immune checkpoint expression [24,26], future research needs to be developed to carefully understand the impact of autophagy in this context.

Among the genes involved in signature B, some of them reveal a significant prognostic value in TCGA cohorts. We showed that *ERO1L* gene was preferentially expressed by malignant cells, suggesting an important role of this protein in the growth of cancer cells. *ERO1L* has already been demonstrated to play a critical role in NSCLC, promoting cancer development by modulating cell cycle-related molecules [27]. Moreover, recent reports also mentioned that ERO1L was implicated in anti-tumor immune response, by preventing T cell-mediated immunity and favoring myeloid suppressor cell activation [28,29]. The expression of *ERO1L* gene in malignant cells could explain, at least in part, the reason for which signature B is associated with a bad prognosis, low infiltration of immune cells and high proliferative rates. Similarly, *ATIC* was much more highly expressed in malignant cells and was associated with a significant prognostic value in our univariate analysis. A recent report demonstrated that ATIC facilitates tumor growth and migration by upregulating Myc expression in LUAD [30]. *BIRC5*, an ATG12-ATG5 conjugate interactor has also been found to be expressed predominantly by tumor cells. BIRC5 was associated with a bad prognosis in lung cancers by favoring mitotic cell cycle-related pathways [31]. In some tumors, BIRC5 was also correlated with high immune cells infiltration [31]. In LUAD, *BIRC5* gene was inversely correlated with dendritic cells and CD4+ T cell infiltration, observations that we confirmed in our analysis. In our signature A, only *DRAM1* was preferentially expressed by tumor cells. DRAM1 was associated with p53 and played a critical role in autophagy and apoptosis [32]. However, the biological function of DRAM1 in lung cancer remains controversial. The study by He Q et al. revealed that DRAM1 could be a target of FTSJ1 and promotes cancer progression [33]. More recently, another study showed that DRAM1 inhibits the development of lung tumors by promoting the lysosomal degradation of EGFR [34]. In our analysis, we showed that *DRAM1* was associated with a good prognostic value, suggesting that the expression of DRAM1 in malignant cells could inhibit tumor growth. Our data highlighted the vital role of these genes expressed by malignant cells in tumor development. While our study strongly suggests a correlation between the expression of these genes and the autophagy level in cancer cells, future investigations should be initiated to understand their role in autophagy modulation in the context of tumor growth.

## 5. Conclusions

Taken together, our analysis reveals a novel autophagy-based signature to determine the metabolic and immunologic status of malignant cells in LUAD. Our study helps to understand the processes involved in LUAD progression and could be useful for therapeutic intervention in NSCLC patients.

## Figures and Tables

**Table 1:** Main information regarding our four datasets. NA means that we do not have the data. TME signifies tumor microenvironment.

Datasets Source	Platform	Samples Types	Subtype	Stage	Number of Samples
TCGA:	Illumina	All TME	LUAD + LUSC	I/II: 879	1129
LUNG	RNAseq			III/IV: 250	
TCGA:	Illumina	All TME	LUAD	I/II: 879	576
LUAD	RNAseq			III/IV: 438	
GEO:	GPL17553	All TME	LUAD	I/II: 138	226
GSE31210	Illumina Hiseq 2000				
GEO:	GPL17553	Sorted	LUAD	NA	21–23
GSE111907	Illumina Hiseq 2000	Cells			
GEO:	GPL16791	Single	LUAD	NA	8 patients (18,124 cells)
GSE123904	Illumina HiSeq 2500	Cells			
GEO:	GPL10558	A549	LUAD cells line	NA	12
GSE73158	Illumina HumanHT-12 V4.0 expression beadchip				

## Data Availability

Data supporting the reported results can be obtained from the corresponding author.

## Supplementary Materials

The following supporting information can be downloaded at: https://www.mdpi.com/article/10.3390/cancers14143462/s1, Figure S1: Autophagy gene screening in lung tumors; Figure S2: Autophagy gene screening and cluster analysis in early stages of lung adenocarcinoma. Figure S3: Autophagy gene screening and cluster analysis in advanced stages of lung adenocarcinoma. Figure S4: Analysis of autophagy genes expression in tumor cells subtypes. Figure S5: Analysis of autophagy genes expression in tumor cells subtypes. Table S1: Statistical report for Figure S3A. Table S2: Statistical report for Figure 5B. Table S3: Statistical report for Figure 5C. Table S4: Statistical report for Figure S3B. Table S5: Statistical report for Figure 5E.
