# Supplementary material for: Autophagy-Related Gene Signature Highlights Metabolic and Immunogenic Status of Malignant Cells in Non-Small Cell Lung Cancer Adenocarcinoma
Source: Cancers (Basel). 2022 Jul 16;14(14):3462. doi: 10.3390/cancers14143462 (PMC9317787; doi:10.3390/cancers14143462)
Supplement: Supplementary file 1 [file cancers-14-03462-s001.zip › supplementary figures.pdf]

Supplemental Figures

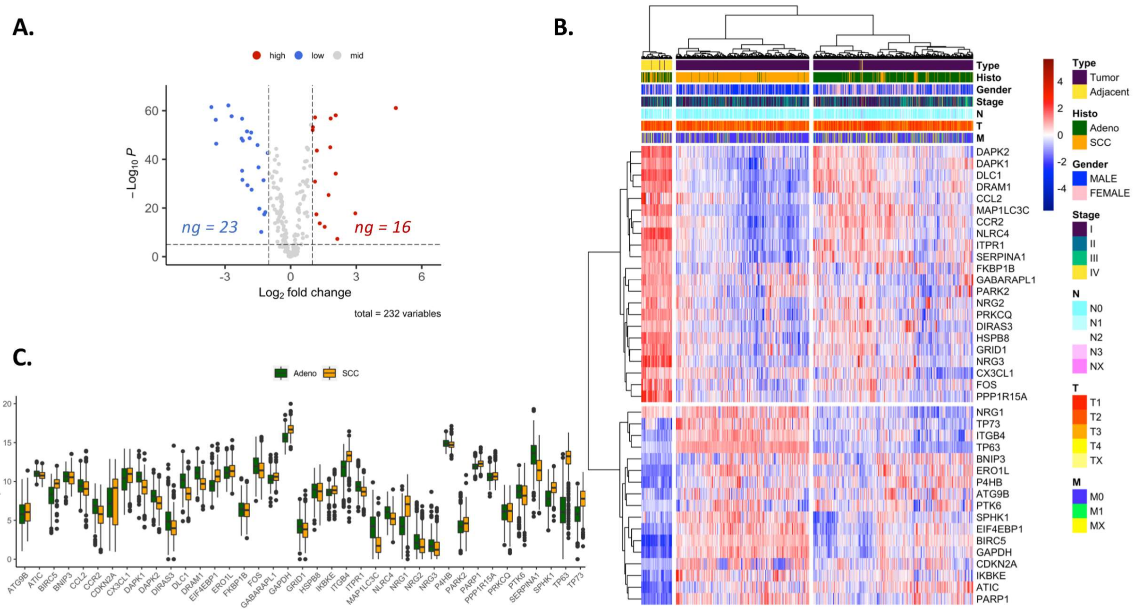

Figure S1. Autophagy gene screening in lung tumors. (A) Differential expression analysis for the human autophagy database (n=232 genes) of TCGA-LUNG patients between tumor (n= 1019) and adjacent tissue (n=110). (**B and C**) Thirty-nine differentially expressed genes of autophagy in TCGA-LUNG datasets. Adeno represents adenocarcinoma and SSC squamous cell carcinoma.

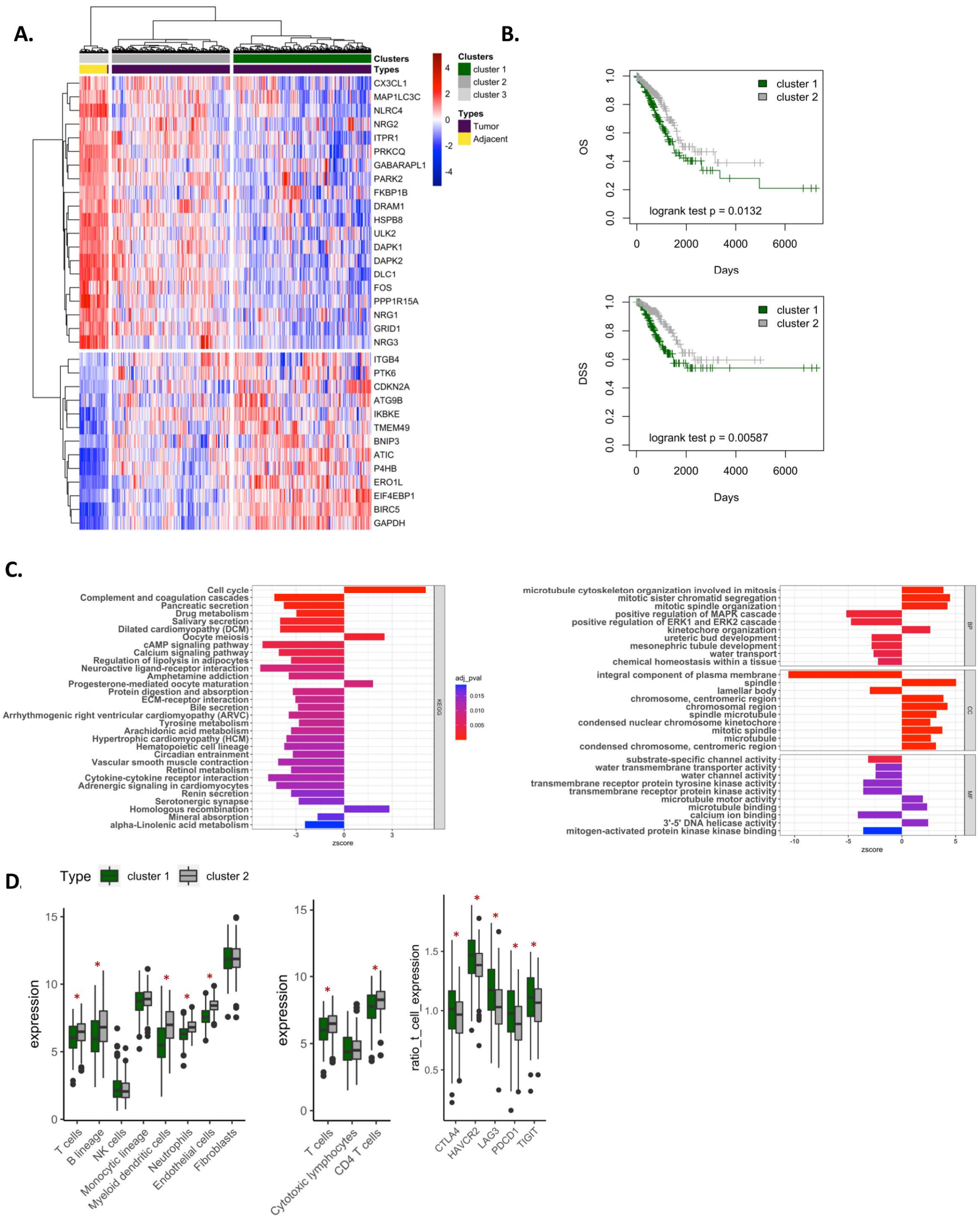

Figure S2. Autophagy gene screening and cluster analysis in early stages of lung adenocarcinoma. (A) Differential expression analysis for the human autophagy database (n=232 genes) of stages I and II TCGA-LUNG datasets (n= 442) samples. (**B**) Kaplan–Meier OS and DSS curve in the stages I and II TCGA-LUNG dataset. (**C**) Representation of the main metabolic pathways modifications between clusters after GO and KEGG enrichment analysis. (**D**) Analysis of immune cells infiltration in clusters. *, p-value < 0.05.

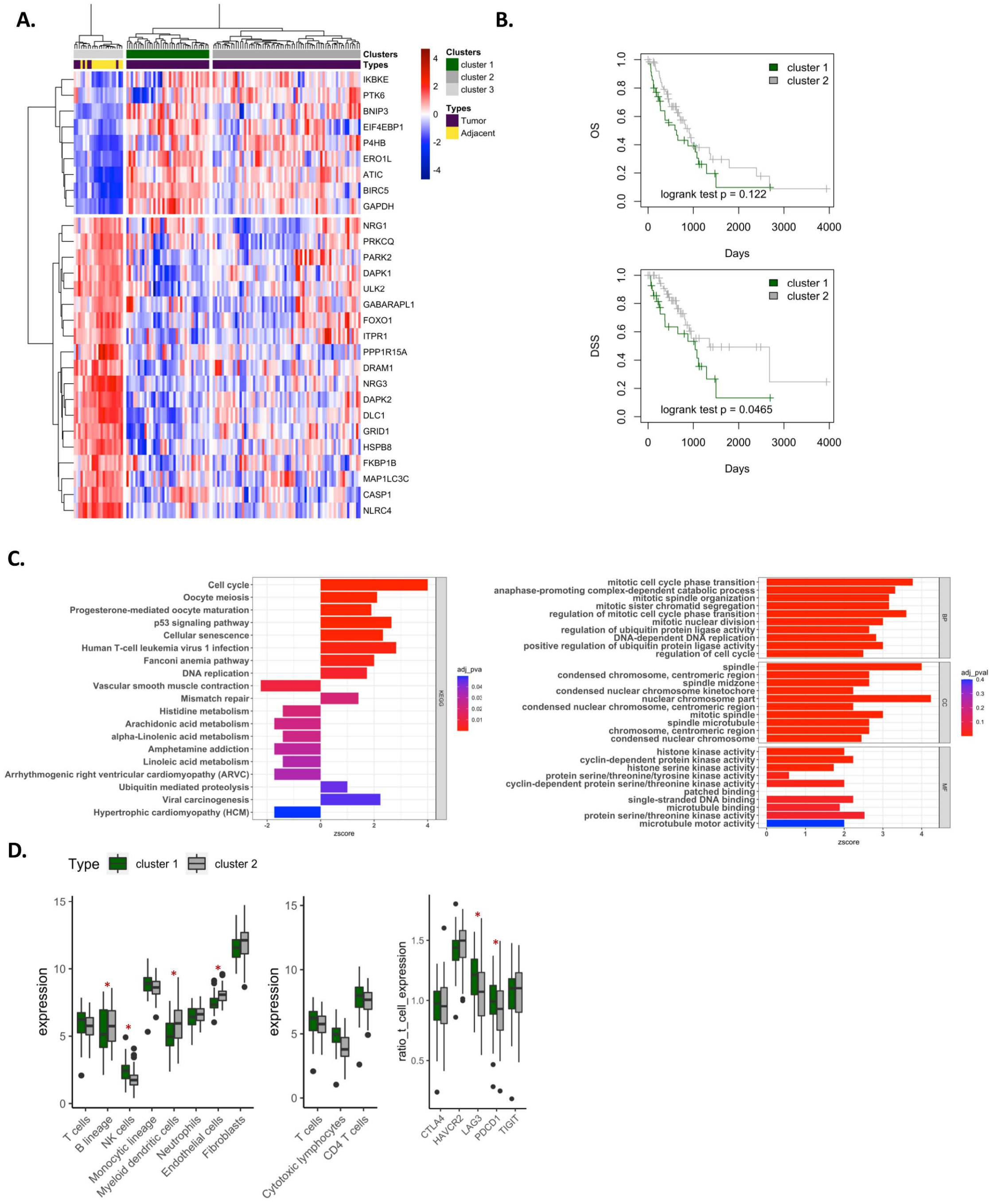

Figure S3. Autophagy gene screening and cluster analysis in advanced stages of lung adenocarcinoma. (A) Differential expression analysis for the human autophagy database (n=232 genes) of stages III and IV TCGA-LUNG datasets (n= 125) samples. (**B**) Kaplan–Meier OS and DSS curve in the stages I and II TCGA-LUNG dataset. (**C**) Representation of the main metabolic pathways modifications between clusters after GO and KEGG enrichment analysis. (**D**) Analysis of immune cells infiltration in clusters. *, p-value < 0.05.

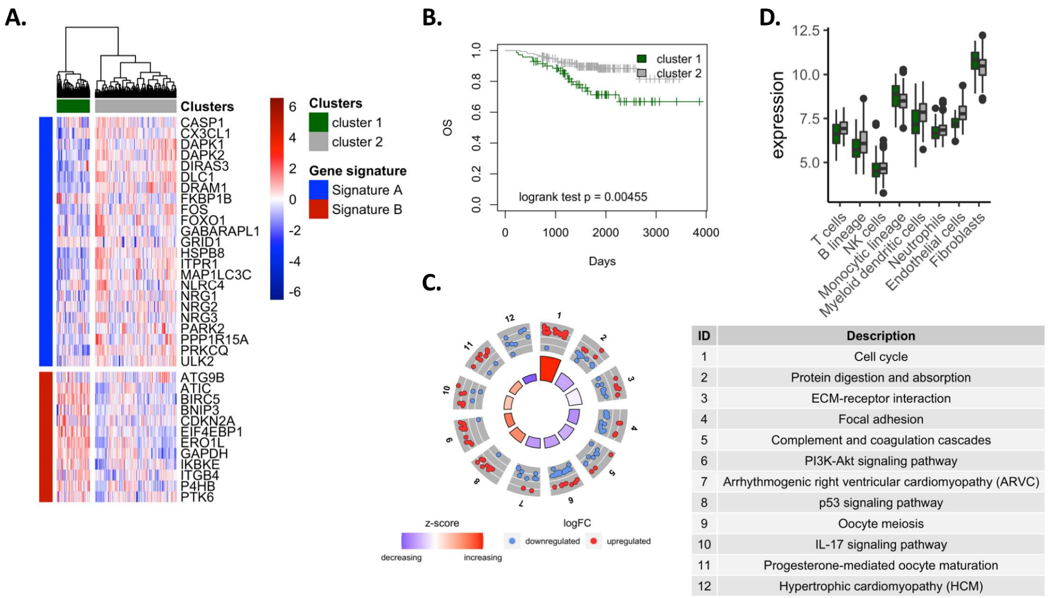

ID	Description
1	Cell cycle
2	Protein digestion and absorption
3	ECM-receptor interaction
4	Focal adhesion
5	Complement and coagulation cascades
6	PI3K-Akt signaling pathway
7	Arrhythmogenic right ventricular cardiomyopathy (ARVC)
8	p53 signaling pathway
9	Oocyte meiosis
10	IL-17 signaling pathway
11	Progesterone-mediated oocyte maturation
12	Hypertrophic cardiomyopathy (HCM)

Figure S4. Autophagy gene screening and cluster analysis in another lung adenocarcinoma cohort. (A) Differential expression analysis for the human autophagy database (n=232 genes) of GSE31210 datasets (n= 226) samples. (**B**) Kaplan–Meier OS curve in the GSE31210 dataset. (**C**) Representation of the main metabolic pathways modifications between clusters after GO and KEGG enrichment analysis. (**D**) Analysis of immune cells infiltration in clusters. *, p-value < 0.05.

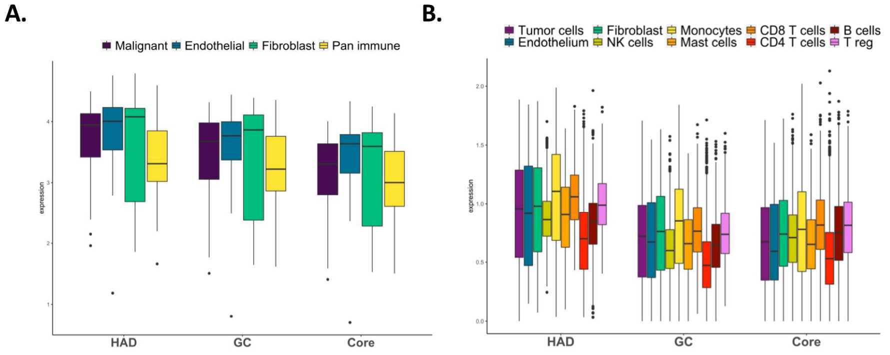

Figure S5. Analysis of autophagy genes expression in tumor cells subtypes. (A, B) Analysis of the human autophagy database (n=232), gene card database (n = 101) and core autophagy database (n = 42) in sorted cells (GSE111907) or single cells (GSE123904) datasets.
